# The passive recipient: Neural correlates of negative self‐view in depression

**DOI:** 10.1002/brb3.2477

**Published:** 2021-12-30

**Authors:** Xiaoyan Wang, Ping Li, Li Zheng, Zhiyuan Liu, Guangcheng Cui, Lin Li, Liangtang Zhang, Qiang Hu, Yu Guo, Lu Wan, Chengchong Li, Yunhui Chen, Zhenghai Sun, Hongsheng Cui, Xin Meng, Yu Si

**Affiliations:** ^1^ School of Psychology Sichuan Normal University Chengdu China; ^2^ School of Psychology and Cognitive Science East China Normal University Shanghai China; ^3^ National Demonstration Center for Experimental Psychology Education East China Normal University Shanghai China; ^4^ Department of Psychiatry Qiqihar Medical University Qiqihar China; ^5^ Shanghai Key Laboratory of Magnetic Resonance East China Normal University Shanghai China; ^6^ Department of Clinical Psychology Qiqihar Mental Health Center Qiqihar China; ^7^ Department of Radiology The Third Affiliated Hospital of Qiqihar Medical University Qiqihar China

**Keywords:** actor/recipient role, attribution, default mode network, depression, negative self‐focused thought

## Abstract

**Introdction:**

Previous studies have argued that people tend to isolate themselves from negative information. This tendency is modulated by the individual's role in social interaction, that is, as an initiative actor (e.g., “I hit Tom”) or a passive recipient (e.g., “Paul hits me”). Depressed patients tend to focus on negative aspects of themselves and cope with situations passively. It is still an open question how the actor/recipient role affects the behavioral and neural responses to self in depression.

**Methods:**

The present study adopted functional magnetic resonance imaging (fMRI) technology to investigate behavioral and neural responses to self (as an actor/recipient) in depressed patients and the matched healthy controls when attributing negative events.

**Results:**

Compared with healthy controls, depressed patients showed more self‐attribution for negative events. Depressed patients showed increased brain activity in the dorsal medial prefrontal cortex (dmPFC) subsystem of the default mode network (DMN) when they played recipient role in self‐related negative events. Activity of the dmPFC subsystem was negatively correlated with depressed patients’ self‐attribution for negative events in recipient condition. While decreased brain activity in the medial temporal lobe (MTL) subsystem was observed in depressed patients when they played the actor or recipient role in self‐related negative events. Activity of the MTL subsystem was negatively correlated with depressed patients’ reaction time when they played recipient role in selfrelated negative events.

**Conclusion:**

These results implicated that depressed patients manifested the negative self‐view. Actor/recipient role affected their activation patterns in the DMN which were different from the healthy controls. The correlation between the abnormal brain activations of the DMN and the behavioral performances might manifest more easily when depressed patients played recipient role in negative events.

## Graphical abstract

1. Depressed patients showed more self‐attribution for negative events than healthy controls.
2. Depressed patients showed increased brain activity in the dmPFC subsystem and decreased brain activity in the MTL subsystem of the default mode network (DMN).
3. Brian activity of the DMN was negatively correlated with depressed patients' level of self‐attribution or reaction time when “self” was the passive recipient of the negative events.
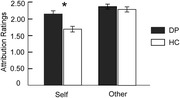

## 1 INTRODUCTION

Depression is a disabling disorder that affects how individuals understand the self and interpersonal situations (Kupferberg et al., 2016). Numerous studies have demonstrated that people experiencing depression exhibit negative self‐focused thought, which is characterized by feelings of worthlessness, self‐blame, and paying close attention to negative aspects of oneself (Korn et al., 2014; Philippi et al., 2018). Critical evidence of negative self‐focused thought has been found in individuals performing an attribution decision task (Hao et al., 2015; Seidel et al., 2012). In these studies, participants were presented with positive and negative self‐related interpersonal events, and were asked to make an attribution in each event. Results showed that depressed patients attributed fewer positive events and more negative events to themselves compared with healthy controls (Hao et al., 2015; Seidel et al., 2012).

Although previous studies have revealed the neural responses to the emotional self in depression, numerous questions remain to be explored. One of them is the role that “self” plays in the interpersonal events. It is well known that, people often play the role of an initiative actor (e.g., “I hit Tom”) or a passive recipient (e.g., “Paul hits me”) in interpersonal events (Wang et al., 2015, 2021; Wang, Zheng et al., 2017). As a recipient, the individual passively receives an uncontrollable action in a social situation. The uncontrollable situation is likely to induce an individual to experience helplessness and change one's self cognition (Billings et al., 1983; Young & Allin Jr, 1986). For example, previous studies have identified that, compared with the role of an actor, individuals were likely to attribute more negative events to themselves and have longer reaction times when they played the role of recipient (Malle, 2006; Wang et al., 2015, 2021; Wang, Zheng et al., 2017). In other words, people experienced more feelings of self‐blame when they played the role of recipient in negative social situations. These feelings from being a recipient were like those of people with depression. Therefore, we predicted that depressed people would attribute more negative events to themselves than healthy controls, and this would be more likely to occur when the individuals played the role of recipient in interpersonal events.

Neuroimaging studies have identified that depression is associated with abnormalities in the default mode network (DMN), especially while people are considering whether negative personality traits or events are self‐relevant and reallocating neural resources to downregulate the negative self‐focused thought (Belleau et al., 2015; Hach et al., 2014; Hao et al., 2015; Lemogne et al., 2009, 2010; Philippi et al., 2018; Seidel et al., 2012; Shao et al., 2018; Sheline et al., 2009; Yoshimura et al., 2010; Zhou et al., 2020). In fact, DMN has emerged as a focus of clinical neuroscientific study in depression in recent years. While it is typically considered as a unit, its functional dissection has identified at least three anatomical–functional subsystems (Andrews‐Hanna et al., 2010; Zhou et al., 2020). One is the core subsystem, including the anterior medial prefrontal cortex (amPFC) and the posterior cingulate cortex, which facilitate self‐referential processing; the other is the dorsal medial PFC (dmPFC) subsystem, comprising the dmPFC, temporoparietal junction, lateral temporal cortex, and temporal pole, which is associated with mentalizing and reflecting on the mental states of oneself and others; the third is the medial temporal lobe (MTL) subsystem, consisting of the ventral mPFC, posterior inferior parietal lobule, retrosplenial cortex, parahippocampal cortex, and hippocampal cortex, which is regarded as being involved in episodic or contextual retrieval or judgments regarding oneself in the future. Previous studies have revealed that depressed patients showed abnormally higher activity in the core subsystem and dmPFC subsystem while showing lower activity in the MTL subsystem compared with healthy controls (Belleau et al., 2015; Hach et al., 2014; Lemogne et al., 2009, 2010, 2012; Zhou et al., 2020). However, studies found that when needing to downregulate the heuristic responses in the attribution decision task, depressed patients showed lower activity of the dmPFC subsystem than healthy controls (Grimm et al., 2009; Seidel et al., 2012). Additionally, Hao et al. (2015) found that the inferior parietal lobule which is the part of the MTL subsystem, showed greater activation in depressed patients than in healthy controls when they evaluated negative self‐related events. These inconsistent results regarding the subsystems of the DMN in depressed patients and healthy controls might be due to the fact that most previous studies used a self‐reference task or self‐focused task to examine the brain responses to self in depression (Lemogne et al., 2009, 2010; Philippi et al., 2018; Sheline et al., 2009). Although these tasks could detect self‐processing directly, self‐processing does not only involve judging the self‐descriptiveness of trait words or mental states. At various moments in life, we might make self‐evaluations in complex interpersonal situations where we might play the role of an active actor or a passive recipient. Under the circumstances of playing different roles, the subsystem of the DMN might be recruited differently when performing self‐related processing. Therefore, it is necessary to further examine how the actor/recipient role affects emotional self‐processing and the underlying neural mechanisms of the subsystem of the DMN in depressed patients and healthy controls.

In the present study, the role played by the self, that is, actor or recipient, in interpersonal events was manipulated in an attribution decision task. Because people are more sensitive to negative information (Zhu et al., 2012), and depression is generally stimulated by negative events (Hao et al., 2015), we selected negative interpersonal events to construct self‐related and other‐related social interaction contexts. Additionally, in order to effectively detect the subsystem of the DMN and its effect on negative self‐processing in depression, independent component analysis (ICA) was applied to analyze the functional magnetic resonance imaging (fMRI) data. ICA is a data‐driven technique that can discover hidden factors underlying a set of random variables, measurements, or signals (Mckeown et al., 1998). It can identify multiple temporally cohesive and spatially distributed regions of brain activities that represent functional connected networks and unique cognitive processes (Wang, Wu et al., 2017). We expected that depressed patients would exhibit a higher level of self‐attribution for negative events compared with healthy controls and that the negative self‐view would be associated with abnormal activities in the DMN. The abnormal behavioral and neural responses in attribution, as hypothesized above, would be pronounced when a depressed patient was involved in a passive and uncontrollable social situation, that is, “the self” playing the role of recipient in an interpersonal event.

## 2 METHODS

### 2.1 Participants

Forty‐two participants were recruited in the present study. Of them, six depressed patients were excluded from all analyses—four because of excessive head movements, one because of little response to the task, and one who gave the same responses in all trials. The remaining 36 participants included in the data analysis consisted of 18 depressed patients and 18 healthy controls. The two groups of participants were matched for gender, age, and educational level. All participants were native Chinese speakers and right‐handed as assessed by the Edinburgh Inventory (Oldfield, 1971). The study was approved by the Research Ethics Committee of East China Normal University and Qiqihar Medical School, and was conducted in accordance with the Declaration of Helsinki. Written informed consent was obtained from each participant before the experiment. All participants were paid 200 RMB (≈$32) for their participation.

Depressed patients were recruited from the Qiqihar Mental Health Center. The patients were diagnosed as having depression if they met the diagnostic and statistical manual of mental disorders (the fourth edition) (DSM‐IV) criteria for major depression and being in a major depressive episode. The exclusion criteria for the depressed patients included co‐morbid psychiatric illness, substance abuse within 6 months of screening, prior electroconvulsive therapy treatments, and a history or current symptoms of psychosis or mania. The severity of affective symptoms was assessed 1−7 days prior to scanning with the 21‐item Beck Depression Inventory (Beck et al., 1961) and the 17‐item Hamilton Depression Rating Scale (Hamilton, 1960). All but four of the depressed patients were taking psychotropic medication at the time of testing. Healthy controls were recruited from the local area (Qiqihar) through poster advertisements, and were free of psychiatric illness or any significant medical conditions. The exclusion criteria for both groups were any neurological disorder or contraindication for fMRI, a history of brain injury, and current pregnancy. Additionally, all participants completed the Positive and Negative Affect Schedule (Watson et al., 1988) and the Rosenberg Self‐Esteem Scale (Rosenberg, 1989). The demographic and clinical characteristics of both groups are listed in Table 1.

**TABLE 1:** Demographic and clinical details of participants

	DP	HC	*p* value
No. of females/males	13/5	9/9	.17
Age (years)	42.14 ± 12.49	39.14 ± 14.39	.51
Years of education	11.61 ± 2.81	12.06 ± 2.65	.25
BDI	29.00 ± 11.40	9.94 ± 8.05	<.001
HAMD	16.44 ± 3.67	n/a	n/a
Age of onset	37.00 ± 11.34	n/a	n/a
Illness duration (years)	0.90 ± 0.55	n/a	n/a
Number of previous episodes	2.88 ± 1.76	n/a	n/a
PANAS positive affect	21.56 ± 4.83	29.72 ± 6.35	<.001
PANAS negative affect	28.72 ± 10.62	18.17 ± 5.58	= .001
RSES	24.89 ± 4.06	31.06 ± 3.81	<.001

Values are means ± standard deviations.

*Abbreviations*: BDI, Beck Depression Inventory; DP, depressed patients; HAMD, Hamilton Depression Rating Scale; HC, healthy controls; PANAS, Positive and Negative Affect Schedule; RSES, Rosenberg Self‐Esteem Scale.

### 2.2 Behavioral paradigm

Participants were presented with 80 sentences describing 40 self‐relevant and 40 other‐relevant negative interpersonal events. For self‐relevant events, “self” was randomly assigned to an actor role (e.g., “I hit Lisa”) or recipient role (e.g., “Mary hits me”), and the self was the target of evaluation (e.g., “How likely is it that I am that kind of person?”). For other‐relevant events (e.g., “Paul hits Tom”, “Ted hits Karl”), both the actor (e.g., “Paul”) and recipient (e.g., “Karl”) would become the targets of evaluation separately (e.g., “How likely is it that Paul/Karl is that kind of person?”). Thus, these stimuli can be categorized into four conditions: actor‐self (AS), actor‐other (AO), recipient‐self (RS), and recipient‐other (RO).

Participants were asked to complete 80 trials in the scanner. In each trial, participants were presented simultaneously with a one‐sentence interpersonal event, an evaluation question, and a 4‐point scale (1 = very unlikely, 2 = moderately unlikely, 3 = moderately likely, 4 = very likely). They were asked to imagine the event happening to them or to others and make an evaluation within 8 s by pressing the corresponding button. Each trial was jittered with inter‐stimuli intervals from 200 to 1000 ms, during which a black fixation cross was presented against a white background. All trials were randomly intermixed and equally divided across two runs.

### 2.3 Image acquisition and preprocessing

All images were collected on a 3T GE Discovery MR750 scanner at the Qiqihar Mental Health Center. Functional images were acquired with an EP∖GR sequence oriented to the AC‐PC (repetition time = 2000 ms, echo time = 30 ms, field of view = 240, 35 slices, slice thickness = 3.5 mm, matrix size = 128 × 128, flip angle = 90^o^) line. A high‐resolution fast‐spoiled gradient recall T1‐weighted image was also acquired from each participant (repetition time = 8.3 ms, echo time = 3.228 ms, matrix size = 256 × 256, 176 slices, slice thickness = 1.2 mm, field of view = 256 mm).

Data analyses were conducted with SPM8 (Wellcome Department of Cognitive Neurology, London). Preprocessing included discarding the first five functional images to allow for scanner equilibrium effects. The remaining 450 functional images were corrected for the delay in slice acquisition and were spatially realigned to the first image. The individual T1‐weighted, 3D structural image was co‐registered to the mean EPI image generated after realignment. The co‐registered structural image was then segmented into gray matter (GM), white matter (WM), and cerebrospinal fluid (CSF) using a unified segmentation algorithm. The functional images after slice timing and realignment procedures were spatially normalized into the Montreal Neurological Institute (MNI) space (resampled at 2 × 2 × 2 mm^3^ voxels) using the normalization parameters from T1 image estimated during unified segmentation, and then spatially smoothed with a Gaussian kernel of 8‐mm full‐width at half‐maximum. A high‐pass filter with a cutoff period of 128 s was applied.

### 2.4 Imaging data analysis

#### 2.4.1 Overview

ICA was used to identify multiple temporally cohesive and spatially distributed regions of brain activities that represent functionally connected networks (Jafri et al., 2008; Kim et al., 2009). To diminish the computational load and to avoid matching components from one group to another, we utilized Group ICA with Infomax to analyze the multi‐subject fMRI data after preprocessing. After that, we conducted statistical analysis of spatial maps and component time courses to select components of interest. The components that passed the three criteria were then subjected to group comparison at the voxel level to identify differences in brain activities between the HC and DP groups. Finally, correlation analyses were conducted to discover the relationship between one's behavioral performance and brain activity. The details of the analyses are as follows.

#### 2.4.2 Group ICA

Group ICA was performed using the GIFT toolbox. Data from the DP and HC groups were equally entered into a joined group ICA. First, data were reduced and compressed using principal component analysis. Second, 20 ICA components (the default value) were estimated since this number offers a good trade‐off between preserving a massive amount of information in the data and reducing the size of the datasets. Third, group spatial ICA was conducted using the Infomax algorithm, which is one of the commonly used ICA algorithms.

#### 2.4.3 The GLM matrix design

The GLM design matrix included the four experimental conditions (i.e., AO, AS, RO, and RS) modeling the attribution task, a fixation condition modeling the baseline, six regressors modeling movement‐related variance, and one regressor modeling the overall mean. For the four experimental conditions of interest, we chose the onset of the stimulus as the onset time point and the RT from the stimulus onset to button press as the duration (epoch with variable time length).

#### 2.4.4 Statistical analysis of spatial maps and component time courses

There were three steps to select the components of interest. First, each component spatial map was correlated with prior probabilistic maps of gray matter (GM), white matter (WM), and CSF within a standardized brain space offered by the MNI templates in SPM8 (Jafri et al., 2008). Based on previous work, the threshold of spatial correlations for CSF was set at *r* ^2 ^< 0.05, *r* ^2 ^< 0.02 for WM, and *r* ^2^ > 0.05 for GM (Kim et al., 2009; Wang, Wu et al., 2017). Components that satisfied these criteria were deemed meaningful and were subjected to the next step. Second, a regression was performed on the ICA time courses with the GLM design matrix, producing a series of beta weights that illustrated the degree to which the component was modulated by the tasks that were related to the fixation baseline. The beta weights associated with the experimental conditions (AO, AS, RO, and RS) for each component underwent a one‐sample *t*‐test for the HC and DP groups separately. Third, a two‐sample *t‐*test was used to determine whether the beta weights produced by the regression showed significant group difference under any of the four experimental conditions (*p* < .05). The components that passed the three criteria were regarded as valid for further analysis.

#### 2.4.5 The group comparison of ICA components

The remaining components in the selection were then subjected to a one‐sample *t* test and a two‐sample *t* test at the voxel level. These analyses were performed using the GIFT toolbox of SPM stats to find the different levels of brain activities between the HC and DP groups. The one‐sample *t*‐test was implemented among all participants for these components to determine whether the beta weights statistically differed from zero. Subsequently, a two‐sample *t‐*test was conducted to identify differences in brain activities between the HC and DP groups (AlphaSim corrected, *p* < .05, 164 contiguous voxels, with an underlying voxel level of *p* < .01 uncorrected).

#### 2.4.6 Correlations between behavioral performance and brain activity

Finally, in order to determine how these brain activities were regulated by the task, we calculated separately the correlations among beta weights, participants’ attribution ratings, and the corresponding reaction times in the experimental conditions.

## 3 RESULTS

### 3.1 Behavioral performance

#### 3.1.1 Attribution ratings

**FIGURE 1:**
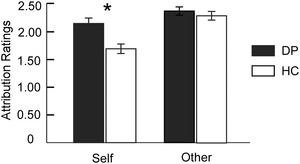
Attribution ratings in the four conditions. Attribution ratings of depressed patients (DP) for self‐related negative events were higher than healthy controls (HC), while there was no difference between the two groups in ratings for other‐related negative events

A 2 (Group: DP vs. HC) × 2 (Target: self vs. other) × 2 (Role: actor vs. recipient) repeated measures ANOVA revealed that participants’ attribution ratings were characterized by a main effect of Target [*F* (1, 34) = 30.78, *p *< .001], and a significant interaction between Group and Target [*F* (1, 34) = 6.45, *p* = .02]. There were no other main effects or significant interactions (all *F* < 3.33, all *p *> .08). Further simple effect analysis revealed that, for self‐related negative events, depressed patients’ attribution ratings were higher than those of healthy controls [*F* (1, 34) = 6.35, *p* = .02]. In contrast, for other‐related negative events, there was no difference in attribution rating between the two groups [*F* (1, 34) = 0.29, *p* = .60] (Figure 1).

#### 3.1.2 Reaction times

A 2 (Group: DP vs. HC) × 2 (Target: self vs. other) × 2 (Role: actor vs. recipient) repeated measures ANOVA revealed that participants’ reaction times (RTs) (Table 2) were characterized by a main effect of Role [*F* (1, 34) = 8.64, *p* = .006], and a significant interaction between Group and Role [*F* (1, 34) = 12.46, *p* = .001]. There were no other main effects or significant interactions (all *F* < 2.75, all *p *> .11).

**TABLE 2:** Participants’ reaction times (ms) to self and other when the evaluation target played the role of actor or recipient in negative interpersonal events (M ± SD)

	DP		HC	
	Self	Other	Self	Other
Actor	3000.84 ± 1253.69	3200.25 ± 1294.52	2703.33 ± 849.81	2850.49 ± 1225.52
Recipient	3059.47 ± 1202.06	3202.24 ± 1190.04	2441.65 ± 932.11	2447.72 ± 920.13

*Abbreviations*: DP, depressed patients; HC, healthy controls.

For the interaction between Group and Role, further simple effect analysis revealed that healthy controls responded slower in actor condition than in recipient condition, *F* (1, 17) = 21.10, *p* < .001, while there was no significant difference between the two conditions in depressed patients, *F* (1, 17) = 0.17, *p* = .68. Additionally, we found that depressed patients’ responding were marginally slower than healthy controls in recipient condition [*F* (1, 34) = 3.86, *p* = .05] but not in actor condition [*F* (1, 34) = 0.76, *p* = .39].

### 3.2 fMRI results

#### 3.2.1 Independent component analysis

Five components, that is, components 5, 8, 15, 18, and 19, passed the three selection criteria (Table 3). Because there was no contiguouos voxel survived in Component 8 at the threshold that used in the present study (AlphaSim corrected, *p* < .05, 164 contiguous voxels, with an underlying voxel level of *p* < .01 uncorrected), the remaining four components were identified in the fMRI results (Table 4). A two‐sample *t*‐test found that Component 15 showed significantly increased beta weights for depressed patients compared with healthy controls only under the RS condition, *t* = 3.24, *p* = .003. Component 5 showed significantly decreased beta weights for depressed patients compared with healthy controls only under the RS condition, *t* = 2.40, *p* = .02. Additionally, we found that Components 18 and 19 showed significantly decreased beta weights for depressed patients compared with healthy controls only under the AS condition (C18: *t* = −2.30, *p* = .03; C19: *t* = −2.24, *p* = .03). There was no other significant difference between the DP and HC groups under other conditions, all *t < *1.98, all *p > *.06 (the details can be seen in Table 3 and Tables A1, A2, A3 in the Appendix).

**TABLE 3:** Results of the two‐sample t test on beta values (M ± SD) between HC and DP groups under self‐related conditions (performed in the GIFT)

	AS			RS		
Components	DP	HC	*t*	DP	HC	*t*
1	0.80 ± 0.71	0.66 ± 0.80	0.54	0.50 ± 0.94	0.83 ± 0.85	−1.09
2	−1.12 ± 1.46	−0.52 ± 1.18	−1.34	−0.77 ± 1.23	−1.10 ± 1.09	0.87
3	1.36 ± 1.09	1.11 ± 1.12	0.69	0.78 ± 1.02	1.58 ± 1.40	−1.98
4	0.95 ± 1.02	0.56 ± 0.88	1.21	0.56 ± 1.01	0.54 ± 0.88	0.08
5	0.55 ± 1.29	0.85 ± 1.24	−0.71	0.20 ± 0.58	1.01 ± 1.31	−2.40*
6	−1.03 ± 1.37	−0.69 ± 1.14	−0.81	−0.98 ± 0.87	−0.85 ± 1.26	−0.35
7	−0.46 ± 1.30	−0.36 ± 0.69	−0.29	−0.49 ± 1.24	−0.39 ± 0.62	−0.30
8	−0.11 ± 1.02	−0.14 ± 0.97	0.11	0.30 ± 0.43	−0.42 ± 1.06	2.65*
9	−0.09 ± 0.69	0.12 ± 0.79	−0.86	−0.02 ± 0.67	0.07 ± 0.83	−0.35
10	1.22 ± 1.61	0.78 ± 1.10	0.97	0.67 ± 0.67	1.05 ± 1.36	−1.06
11	1.82 ± 2.12	1.84 ± 1.42	−0.03	1.60 ± 1.38	1.94 ± 1.50	−0.69
12	0.13 ± 1.45	0.12 ± 1.32	0.03	0.09 ± 1.29	−0.10 ± 1.06	0.48
13	0.26 ± 1.58	0.23 ± 0.93	0.08	0.25 ± 1.10	0.10 ± 1.13	0.38
14	0.04 ± 1.11	0.08 ± 0.94	−0.13	−0.17 ± 0.84	0.08 ± 1.03	−0.78
15	−0.07 ± 0.68	−0.43 ± 0.81	1.42	0.09 ± 0.63	−0.77 ± 0.95	3.24**
16	1.18 ± 1.63	0.80 ± 1.07	0.83	0.81 ± 1.18	0.96 ± 1.18	−0.39
17	1.19 ± 1.02	0.98 ± 1.17	0.59	1.14 ± 0.85	0.79 ± 1.28	0.98
18	−0.34 ± 0.65	0.39 ± 1.18	−2.30*	0.07 ± 0.63	0.24 ± 1.02	−0.61
19	−1.04 ± 1.34	−0.04 ± 1.35	−2.24*	−0.74 ± 0.99	−0.35 ± 1.36	−0.97
20	−0.04 ± 0.65	0.31 ± 0.96	−1.29	0.11 ± 0.92	−0.15 ± 0.85	0.90

*Abbreviations*: AS, “self” was the actor of negative event; DP, depressed patients; HC, healthy controls; RS, “self” was the recipient of negative event.

*p < .05, ** *p* < .01.

**TABLE 4:** Differences in brain regions of components 5, 8, 15, 18, and 19 based on a two‐sample *t*‐test (AlphaSim corrected)

			MNI				
Components	Regions	Brodmann areas	*X*	*Y*	*Z*	*t* value	Voxels
5	Superior temporal gyrus	22	60	−34	6	3.53	169
		22	54	−32	4	3.37	
		21	64	−24	0	3.03	
8	No activation survived						
15	Dorsal medial prefrontal cortex	32	−18	48	20	4.28	254
		32	−12	52	24	4.23	
		32	−14	54	22	4.07	
18	Ventral medial prefrontal cortex	47	−28	48	0	3.33	168
	*Orbital frontal cortex*	11	−24	48	−10	2.88	
19	Postcentral gyrus	3	38	−30	42	4.32	432
	*Middle cingulate cortex*	23	−6	−46	34	3.29	315
	*Hippocampus*	37	−24	−36	−2	4.27	181

To determine which networks these components belonged to, the brain regions involved in these components indicating the differences between the two groups were identified as shown in Table 4. Brain images of Components 5, 15, 18, and 19 were compared with 90 functional regions of interest (fROIs) atlases created by Shirer et al. (2012). All brain regions were involved in the DMN, so these components were confirmed to be involved in the DMN. Moreover, according to the identified functional dissection in the DMN (Andrews‐Hanna et al., 2010; Zhou et al., 2020), Component 15 was involved in the dmPFC subsystem, and Components 5, 18, and 19 were involved in the MTL subsystem.

#### 3.2.2 Correlation results of behavioral performance and brain activity

To further identify how these group differences in brain activities in each component were regulated by the corresponding experimental condition in the task, correlation analyses were conducted. The results showed that the beta weights of the dmPFC subsystem (i.e., Component 15) in the RS condition were negatively correlated with individuals’ attribution ratings in the DP group (*r *= −0.50, *p* = .03) but not in the HC group (*r *= 0.30, *p* = .22).

The beta weights of the MTL subsystem (i.e., Component 5) in the RS condition negatively correlated with individuals’ RT in the DP group (*r *= −0.55, *p* = .02) but not in the HC group (*r *= −0.06, *p* = .81). There was no other significant correlation between the beta weights of these components and behavioral performance in either the HC or the DP group in the four experimental conditions (all *r* < 0.44, all *p* > .07).

## 4 DISCUSSION

The present study investigated how the actor or recipient role affected the behavioral and neural responses of depressed patients and healthy controls when attributing negative interpersonal events. At the behavioral level, depressed patients made more self‐attributions for negative events than healthy controls. At the neural level, greater brain activity in the dmPFC subsystem of DMN was found in depressed patients compared with healthy controls when they passively received negative self‐related events (i.e., the RS condition). Moreover, the greater the activity in the dmPFC subsystem of a depressed patient, the less self‐attribution for negative events was observed in the RS condition. Additionally, we found less activity in the MTL subsystem in depressed patients than in healthy controls when they played the actor or recipient role in self‐related negative events. But depressed patients only showed a negative correlation between MTL activity and reaction time in RS condition.

A central feature of depressed patients is having elevated feelings of self‐blame and paying more attention to negative aspects of themselves (Korn et al., 2014; Philippi et al., 2018). Our results showed that depressed patients endorsed more negative events for themselves compared with healthy controls. Along with prior studies (Hao et al., 2015; Seidel et al., 2012), these results indicated that when exposed to self‐related negative situations, depressed patients were more likely to blame themselves than healthy controls. At the neural level, greater brain activity in the dmPFC subsystem of the DMN was found in depressed patients compared with healthy controls only when the “self” played the role of recipient in negative events. Previous studies have argued that healthy controls tend to isolate themselves from negative events, while making self‐attribution for negative events is a heuristic response for depression (Seidel et al., 2012). Self‐reappraisal has been confirmed to helpful to lessen one's heuristic response through consuming more cognitive resources (Beer & Hughes, 2010; Seidel et al., 2012). Key regions within the dmPFC subsystem, such as the dmPFC, are widely associated with self‐focus, self‐related reappraisal, mentalizing, and cognitive control (Korn et al., 2012; Lemogne et al., 2012; Northoff et al., 2004; Seidel et al., 2012). Thus, we thought that greater activity of the dmPFC subsystem in the present study might be associated with self‐reappraisal. This speculation was confirmed by our correlation analyses. That is, the greater the activity in the dmPFC subsystem of a depressed patient, the less the self‐attribution for negative events was observed in RS condition. These results suggested that self‐reappraisal and the engagement of the dmPFC subsystem might be useful to alleviate the negative self‐attribution in depression when they played recipient role.

In contrast, we found that the MTL subsystem of the DMN was less activated in depressed patients than in healthy controls. Previous studies have argued that these regions are associated with autobiographical memory and information retrieval (Viard et al., 2007; Wais, 2008). However, depression is characterized by disturbance in ways autobiographical memories are represented, recalled, and maintained (Dalgleish & Werner‐Seidler, 2014; Dillon & Pizzagalli, 2018; McDermott & Ebmeier, 2009). One core symptom was the systematic biases in favor of negative material. In the present study, making attribution ratings for self‐related negative events might be associated with information retrieval from one's autobiographical memory. Few cognitive resources might be consumed to retrieval self‐related negative events from depressed patients’ autobiographical memory. Therefore, the MTL subsystem might be engaged less in retrieving such information in depression compared with healthy controls, no matter which role was played. Moreover, we found that depressed patients made marginally slower responses than healthy controls in recipient condition. And the lower the activity of the MTL subsystem of depression, the longer the RT was observed in the RS condition. These results were consistent with the previous studies that depressed patients have dysfunctional processes of avoidance around personal autobiographical material (Dalgleish & Werner‐Seidler, 2014). These dysfunctions might make depressed patients sucked in negative events and responded slowly, especially when they were passively subjected to negative events.

Consistent with the previous studies on the self‐processing of depression using the attribution rating task, we confirmed that depressed patients manifested negative self‐view and different activation patterns in the DMN from the healthy controls. Importantly, we distinguished the role played by the self in interpersonal events, that is, an actor or a recipient, in the present study. We found preliminarily that actor/recipient role affected depressed patients’ activations of the DMN. The correlation between the abnormal brain activations of the DMN and the behavioral performances might manifest more easily when depressed patients played recipient role in negative events. However, there were limitations to the present study that should be acknowledged. All but four of the depressed patients were receiving antidepressant medication at the time of the study, which meant that the results might be confounded by medication status. Additionally, the sample size of the present study may have been too small to provide strong evidence to illustrate the neural mechanism of how the actor/recipient role affects one's emotional self‐processing. Therefore, caution should be applied when interpreting these findings, and further research is needed to better understand the effect of the actor/recipient role on self‐cognition in depressive disorders.

## 5 CONCLUSION

To the best of our knowledge, this was the first study that explored the underlying neural correlates of how the actor/recipient role affects negative self‐processing in depressive disorders. Compared with healthy controls, depressed patients exhibited greater activity in the dmPFC subsystem and less activity in the MTL subsystem of the DMN. Especially when depressed patients were passively subjected to negative interpersonal events, brain activity in the dmPFC subsystems was negatively correlated with self‐attribution for negative events. These results suggested that increased brain activity in the dmPFC subsystem and decreased brain activity in the MTL subsystem of the DMN could be a potential biomarker for depression. The results also highlighted the importance of exploring the effect of the actor or recipient role on self‐processing in social situations.

## CONFLICT OF INTEREST

The authors report no conflict of interest.

### PEER REVIEW

The peer review history for this article is available at https://publons.com/publon/10.1002/brb3.2477

## Data Availability

The data that support the findings of this study are available from the corresponding author upon reasonable request.

## APPENDIX A

**TABLE A1:** Beta values (M ± SD) of each condition on 20 components in healthy control group, and results of the one‐sample *t*‐test under each condition (performed in GIFT)

	Conditions							
	*AO*		*AS*		*RO*		*RS*	
Components	*β*	*t*	*Β*	*T*	*β*	*t*	*β*	*t*
1	0.41 ± 0.84	2.07	0.66 ± 0.80	3.51**	0.59 ± 0.77	3.23**	0.83 ± 0.85	4.13***
2	−0.94 ± 0.97	−4.10***	−0.52 ± 1.18	−1.88	−0.76 ± 0.59	−5.46***	−1.10 ± 1.09	−4.30***
3	1.30 ± 0.90	6.14***	1.11 ± 1.12	4.20***	1.38 ± 1.48	3.93**	1.58 ± 1.40	4.80***
4	0.58 ± 1.03	2.40*	0.56 ± 0.88	2.70*	0.67 ± 0.86	3.30**	0.54 ± 0.88	2.59*
5	1.04 ± 1.25	3.52**	0.85 ± 1.24	2.90**	1.00 ± 1.93	2.19*	1.01 ± 1.31	3.26**
6	−0.56 ± 1.16	−2.06	−0.69 ± 1.14	−2.57*	−0.81 ± 1.32	−2.60*	−0.85 ± 1.26	−2.87*
7	−0.14 ± 0.78	−0.77	−0.36 ± 0.69	−2.19*	−0.45 ± 0.56	−3.38**	−0.39 ± 0.62	−2.69*
8	0.03 ± 0.87	0.15	−0.14 ± 0.97	−0.63	−0.13 ± 1.01	−0.52	−0.42 ± 1.06	−1.67
9	0.23 ± 0.62	1.54	0.12 ± 0.79	0.65	−0.02 ± 0.61	−0.17	0.07 ± 0.83	0.35
10	0.61 ± 1.09	2.39*	0.78 ± 1.10	2.99**	0.71 ± 1.41	2.14*	1.05 ± 1.36	3.28**
11	1.88 ± 1.24	6.45***	1.84 ± 1.42	5.49***	2.01 ± 1.89	4.53***	1.94 ± 1.50	5.46***
12	−0.30 ± 1.06	−1.20	0.12 ± 1.32	0.38	−0.07 ± 0.84	−0.35	−0.10 ± 1.06	−0.41
13	−0.19 ± 0.91	−0.90	0.23 ± 0.93	1.03	−0.20 ± 1.15	−0.73	0.10 ± 1.13	0.39
14	0.02 ± 0.59	0.86	0.08 ± 0.94	0.38	−0.07 ± 0.66	−0.46	0.08 ± 1.03	0.32
15	−0.47 ± 1.13	−1.75	−0.43 ± 0.81	−2.23*	‐0.25 ± 0.75	−1.43	−0.77 ± 0.95	−3.47**
16	1.06 ± 1.12	4.03***	0.80 ± 1.07	3.17**	1.17 ± 1.19	4.17***	0.96 ± 1.18	3.46**
17	0.82 ± 1.08	3.23**	0.98 ± 1.17	3.55**	0.92 ± 1.09	3.58**	0.79 ± 1.28	2.61*
18	0.22 ± 1.13	0.83	0039 ± 1.18	1.39	0.27 ± 1.36	0.83	0.24 ± 1.02	1.01
19	−0.49 ± 1.21	−1.73	−0.04 ± 1.35	−0.11	−0.14 ± 1.35	−0.44	−0.35 ± 1.36	−1.09
20	0.21 ± 0.91	0.98	0.31 ± 0.96	1.36	0.40 ± 1.27	1.35	−0.15 ± 0.85	−0.76

*Note*: AO, “other person” was the actor of the other‐related negative event, AS, “self” was the actor of the self‐related negative event; RO, “other person” was the recipient of the other‐related negative event; RS, “self” was the recipient of the self‐related negative event.

**p *< .05, ** *p* < .01, *** *p* < .001.

**TABLE A2:** Beta values (M ± SD) of each condition on 20 components in depressed patient group, and results of the one‐sample t test under each condition (performed in GIFT)

	Conditions							
	*AO*		*AS*		*RO*		*RS*	
Components	*β*	*t*	*β*	*t*	*β*	*t*	*β*	*t*
1	0.52±1.17	1.88	0.80 ± 0.71	4.75***	0.68 ± 1.04	2.77*	0.50 ± 0.94	2.25*
2	−1.03 ± 1.16	−3.78**	−1.12 ± 1.46	−3.23**	−0.80 ± 1.19	−2.84*	−0.77 ± 1.23	−2.65*
3	1.60 ± 1.35	5.02***	1.36 ± 1.09	5.30***	1.58 ± 1.05	6.36***	0.78 ± 1.02	3.24**
4	0.57 ± 1.37	1.76	0.95 ± 1.02	3.92**	0.85 ± 1.12	3.24**	0.56 ± 1.01	2.36*
5	0.66 ± 1.44	1.92	0.55 ± 1.29	1.81	0.55 ± 0.87	2.67*	0.20 ± 0.58	1.44
6	−1.23 ± 2.36	−2.21*	−1.03 ± 1.37	−3.18**	−1.11 ± 1.57	−2.98**	−0.98 ± 0.87	−4.76***
7	−0.40 ± 1.35	−1.25	−0.46 ± 1.30	−1.49	−0.59 ± 1.15	−2.18*	−0.49 ± 1.24	−1.69
8	0.42 ± 1.15	1.54	−0.11 ± 1.02	−0.45	−0.05 ± 0.71	−0.31	0.30 ± 0.43	2.92**
9	0005 ± 1.23	0.17	−0.09 ± 0.69	−0.56	−0.13 ± 0.55	−0.98	−0.02 ± 0.67	−0.12
10	0.85 ± 1.18	3.05**	1.22 ± 1.61	3.22**	0.86 ± 1.20	3.04**	0.67 ± 0.67	4.23***
11	1.81 ± 1.66	4.64***	1.82 ± 2.11	3.65**	1.64 ± 1.65	4.23***	1.60 ± 1.38	4.94***
12	−0.25 ± 1.02	−1.04	0.13 ± 1.45	0.38	−0.22 ± 1.23	−0.76	0.09 ± 1.29	0.28
13	0.01 ± 0.92	0.03	0.26 ± 1.58	0.71	0.29 ± 1.06	1.17	0.25 ± 1.10	0.95
14	0.01 ± 1.68	0.03	0.04 ± 1.11	0.15	−0.03 ± 1.05	−0.11	−0.17 ± 0.84	−0.84
15	−0.23 ± 1.48	−0.65	−0.07 ± 0.68	−0.44	−0.04 ± 0.87	−0.18	0.09 ± 0.63	0.62
16	0.94 ± 0.89	4.45***	1.18 ± 1.63	3.07**	0.63 ± 0.86	3.08**	0.81 ± 1.18	2.91**
17	1.22 ± 1.38	3.74**	1.19 ± 1.02	4.94***	0.94 ± 0.97	4.11***	1.14 ± 0.85	5.73***
18	−0.02 ± 0.81	−0.11	−0.34 ± 0.65	−2.24*	0.09 ± 0.42	0.87	0.07 ± 0.63	0.47
19	−0.88 ± 0.88	−4.23***	−1.04 ± 1.34	−3.29**	−0.52 ± 1.01	−2.19*	−0.74 ± 0.99	−3.14**
20	0.47 ± 1.04	1.93	−0.04 ± 0.65	−0.28	0.33 ± 0.75	1.87	0.11 ± 0.92	0.52

*Note*: AO, “other person” was the actor of the other‐related negative event, AS, “self” was the actor of the self‐related negative event; RO, “other person” was the recipient of the other‐related negative event; RS, “self” was the recipient of the self‐related negative event.

**p *< .05, ** *p* < .01, *** *p* < .001.

**TABLE A3:** Results of the two‐sample t test on beta values (M ± SD) between HC and DP groups under other‐related conditions (performed in GIFT)

	AO				RO			
Components	HC	DP	*t*	*p*	HC	DP	*t*	*p*
1	0.41 ± 0.84	0.52 ± 1.17	−0.31	.76	0.59 ± 0.77	0.68 ± 1.04	−0.30	.77
2	−0.94 ± 0.97	−1.03 ± 1.16	0.26	.79	−0.76 ± 0.59	−0.80 ± 1.19	0.11	.91
3	1.30 ± 0.90	1.60 ± 1.35	−0.77	.45	1.38 ± 1.48	1.58 ± 1.05	−0.47	.64
4	0.58 ± 1.03	0.57 ± 1.37	0.04	.97	0.67 ± 0.86	0.85 ± 1.12	−0.56	.58
5	1.04 ± 1.25	0.66 ± 1.44	0.85	.40	1.00 ± 1.93	0.55 ± 0.87	0.90	.37
6	−0.56 ± 1.16	−1.23 ± 2.36	1.07	.29	−0.81 ± 1.32	−1.11 ± 1.57	0.62	.54
7	−0.14 ± 0.78	−0.40 ± 1.35	0.70	.49	−0.45 ± 0.56	−0.59 ± 1.15	0.48	.64
8	0.03 ± 0.87	0.42 ± 1.15	−1.14	.26	−0.13 ± 1.01	−0.05 ± 0.71	−0.25	.81
9	0.23 ± 0.62	0.05 ± 1.23	0.54	.59	−0.02 ± 0.61	−0.13 ± 0.55	0.53	.60
10	0.61 ± 1.09	0.85 ± 1.18	−0.62	.54	0.71 ± 1.41	0.86 ± 1.20	−0.34	.74
11	1.88 ± 1.24	1.81 ± 1.66	0.14	.89	2.01 ± 1.89	1.64 ± 1.65	0.63	.53
12	−0.30 ± 1.06	−0.25 ± 1.02	−0.14	.89	−0.07 ± 0.84	−0.22 ± 1.23	0.43	.67
13	−0.19 ± 0.91	0.01 ± 0.92	0.66	.52	−0.20 ± 1.15	0.29 ± 1.06	−1.33	.19
14	0.12 ± 0.59	0.01 ± 1.68	0.26	.80	−0.07 ± 0.66	−0.03 ± 1.05	−0.15	.88
15	−0.47 ± 1.13	−0.23 ± 1.47	−0.55	.59	−0.25 ± 0.75	−0.04 ± 0.87	−0.80	.43
16	1.06 ± 1.12	0.94 ± 0.89	0.37	.72	1.17 ± 1.19	0.63 ± 0.86	1.57	.12
17	0.82 ± 1.08	1.22 ± 1.38	−0.96	.35	0.92 ± 1.09	0.94 ± 0.97	−0.06	.95
18	0.22 ± 1.13	−0.02 ± 0.81	0.74	.47	0.27 ± 1.36	0.09 ± 0.42	0.54	.59
19	−0.49 ± 1.21	−0.88 ± 0.88	1.09	.28	−0.14 ± 1.35	−0.52 ± 1.01	0.96	.35
20	0.21 ± 0.91	0.47 ± 1.04	−0.81	.43	0.40 ± 1.27	0.33 ± 0.75	0.21	.83

*Note*: AO, “other person” was the actor of the other‐related negative event; RO, “other person” was the recipient of the other‐related negative event.

*Abbreviations*: DP, depressed patients; HC, healthy controls.
